# A Rare Case of Congenital Plunging Ranulas: Diagnosis With Intraoral and Extraoral Ultrasound and Magnetic Resonance Imaging

**DOI:** 10.7759/cureus.37049

**Published:** 2023-04-02

**Authors:** Udai Chowdhary, Suresh Phatak, Avinash Dhok, Priya Potdukhe

**Affiliations:** 1 Department of Radiodiagnosis, N.K.P. Salve Institute of Medical Sciences and Research Centre & Lata Mangeshkar Hospital, Nagpur, IND

**Keywords:** sublingual swelling, submandibular pseudocyst, submandibular swelling, mucous retention cyst, plunging ranula, ranula

## Abstract

Ranulas are cystic lesions located in the floor of the mouth. These are “pseudocysts” and are developed due to an obstruction in the sublingual gland. Congenital variants of plunging ranulas are very rare. Here, we report a case of an eight-year-old male child presenting with congenital swelling with an intraoral component as well as extension to the submandibular gland region. The swelling was painless and gradually growing in size.

## Introduction

Ranulas are cystic lesions in the floor of the mouth developed due to obstruction to the outflow of saliva in the sublingual gland [1]. They have derived their nomenclature from the Latin word “Rana” meaning frog, due to their peculiar translucent bluish color resembling the belly of a frog [2]. Due to an obstruction to the outflow of saliva in the sublingual gland, there is proximal dilatation of the sublingual gland duct, followed eventually by its rupture, leading to extravasation of the saliva into surrounding soft tissues. This incites an inflammatory response leading to condensation of connective tissue at the periphery of collection; hence on histopathological examination, ranulas are characteristically devoid of epithelium and endothelium and are also referred to as ‘pseudocysts’ [3]. Ranulas are of two types: 1) simple ranulas and 2) plunging ranulas. Simple ranulas are cystic lesions confined to the floor of the mouth with no extension into the submandibular space. On the other hand, ranulas that extend or plunge beyond the mylohyoid muscle to enter the adjacent spaces like submandibular or parapharyngeal space are known as ‘plunging ranulas’ [2]. In some cases, dehiscence or break in the mylohyoid muscle itself paves way for the extension of cysts into the submandibular region [1]. With a prevalence of 0.2 cases per 1000 individuals, ranulas make up for six percent of all oral cystic lesions [2]. However, congenital ranulas are very rare with a prevalence of 0.79%. The surgical approach while dealing with a cystic lesion of the floor of the mouth is intraoral for the lesions limited to the floor of the mouth and extraoral for submandibular lesions. But in the case of ranulas, regardless of the extension of lesions, the approach is always intraoral excision of the sublingual gland. This makes it crucial to differentiate ranulas from other cystic lesions as the decision of surgical approach changes [4].

## Case presentation

**Figure 1:**
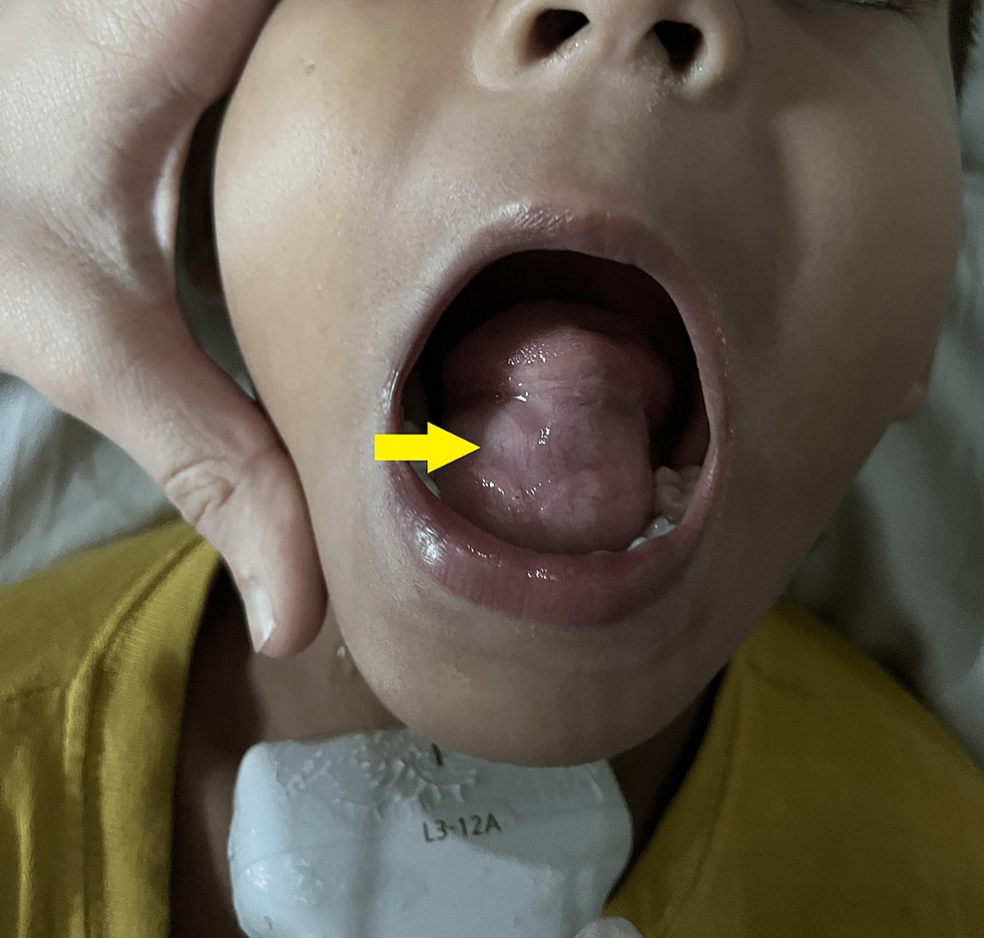
Clinical photograph of sublingual soft tissue swelling (yellow arrow).

An eight-year-old male child presented with a painless and gradually increasing swelling occupying the floor of the mouth (Figure 1) and a submandibular region in the midline since birth. Initially, the patient was asymptomatic but as the sublingual component increased in size it caused lifting of the tongue and speech hindrance. On clinical examination, the swelling had two components: a small submandibular component and a characteristic sublingual component in the floor of the mouth. On palpation, the tongue was fluctuant, freely movable, and non-tender.

Ultrasound

**Figure 2:**
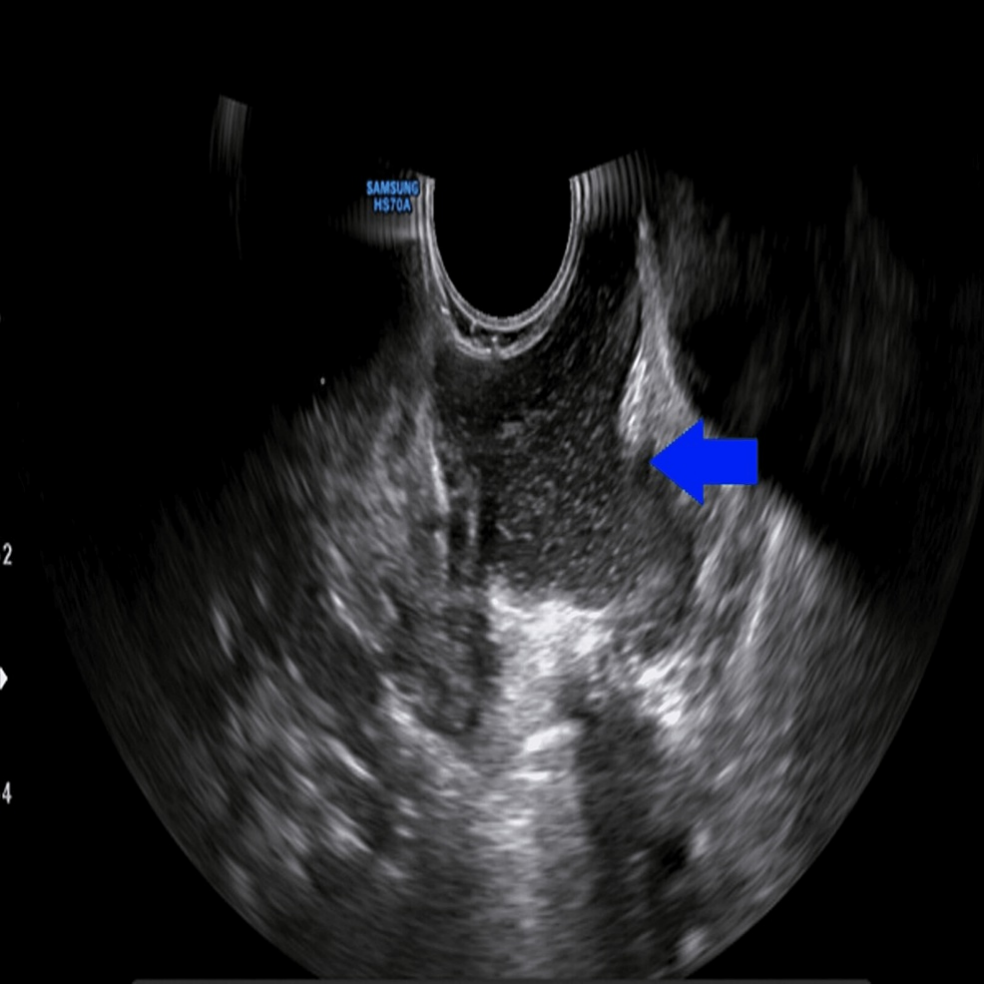
Intraoral B-mode ultrasound showing a sublingual component as a well-defined cystic lesion in the floor of the mouth with multiple internal echoes within it (blue arrow).

**Figure 3:**
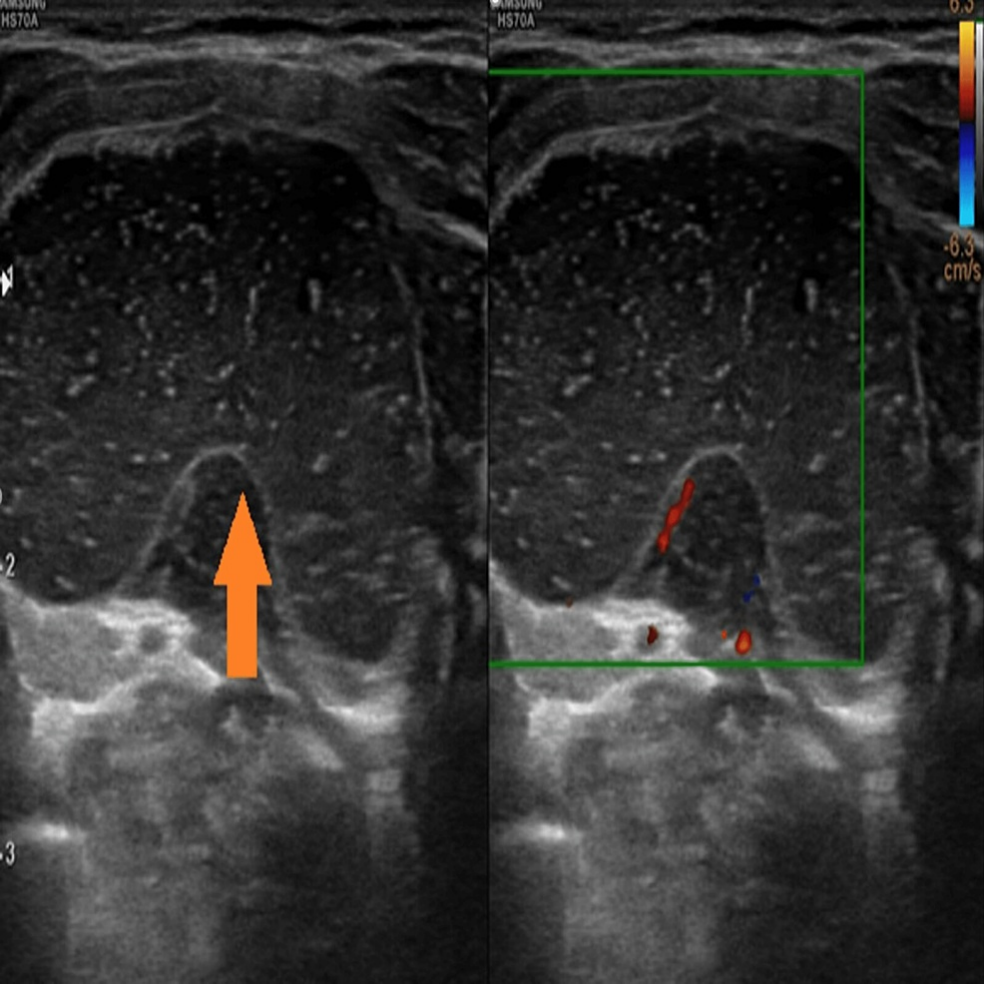
B-mode ultrasound of the submandibular region showing the submandibular component of the ranula (orange arrow) as a morphologically similar well-defined cystic lesion with multiple moving internal echoes within it. No vascularity was demonstrated on color Doppler. No calcification or septation was seen.

**Figure 4:**
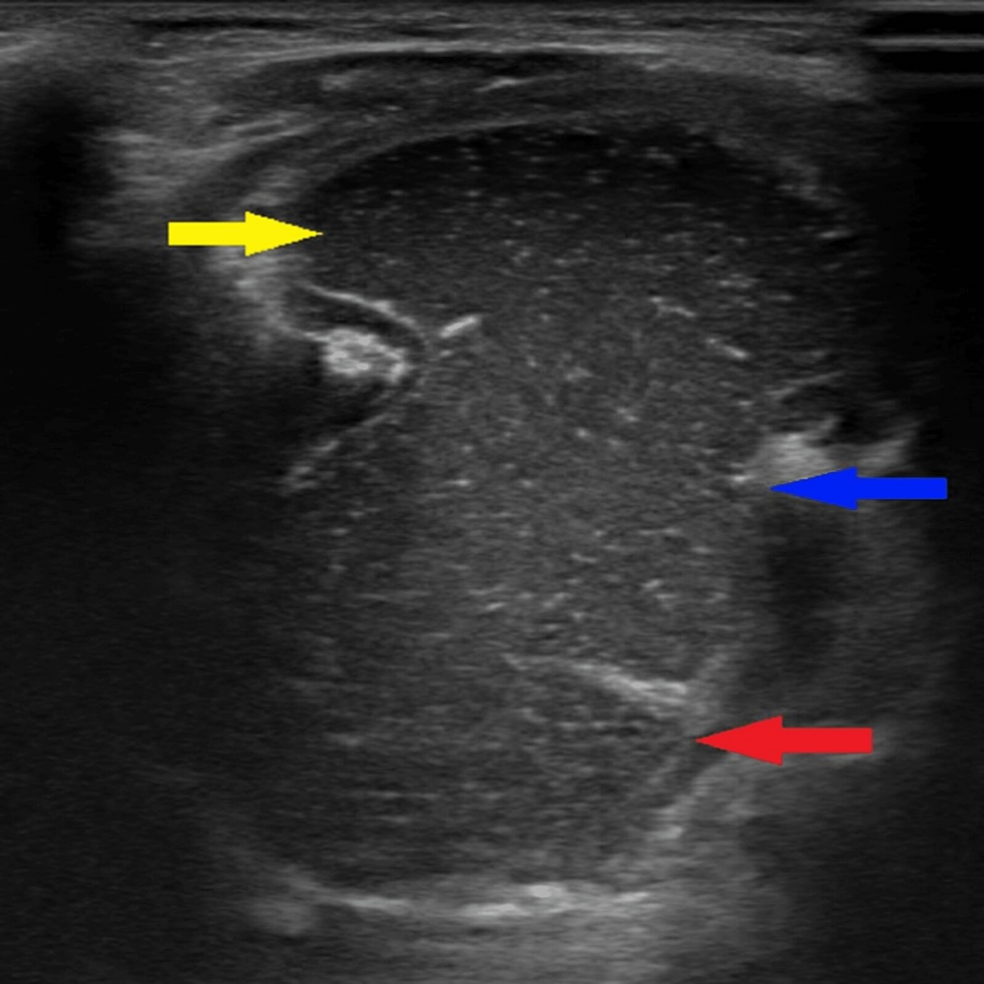
B-mode ultrasound in the oblique sagittal plane showing a sublingual component (yellow arrow) and a submandibular component (red arrow). A defect in the mylohyoid muscle is present through which the ranula is extending from the sublingual to submandibular region (blue arrow).

 A well-defined cystic lesion in the midline with an intraoral component measuring 2.4 x 1.3 cm and a submandibular component measuring 3.2 x 2.8 cm showed multiple moving internal echoes (Figures 2-4). It showed peripheral vascularity on color Doppler. There was no calcification.

 

Magnetic resonance imaging

**Figure 5:**
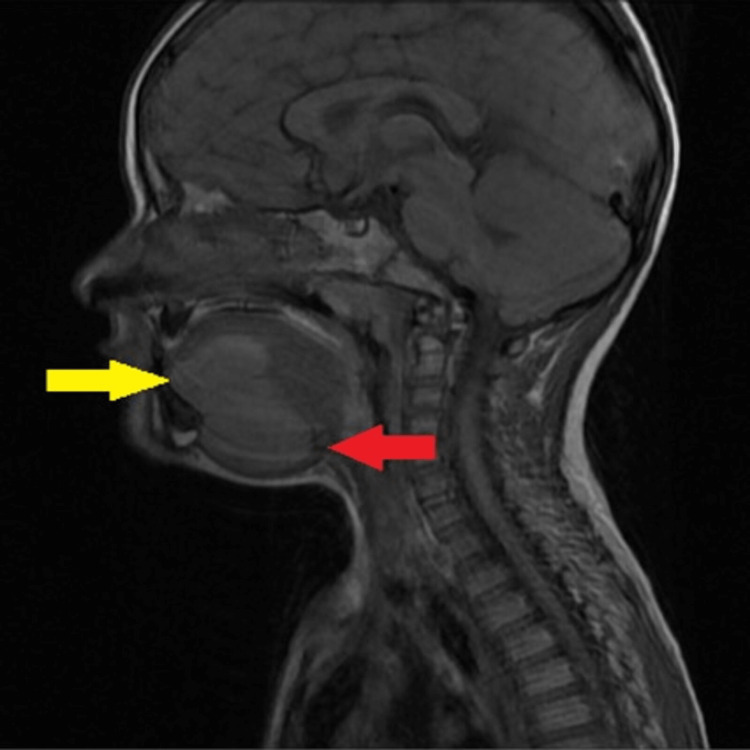
Sagittal T1 weighted image showing a well-defined iso-hypointense lesion with a sublingual component (yellow arrow) and a submandibular component (red arrow).

**Figure 6:**
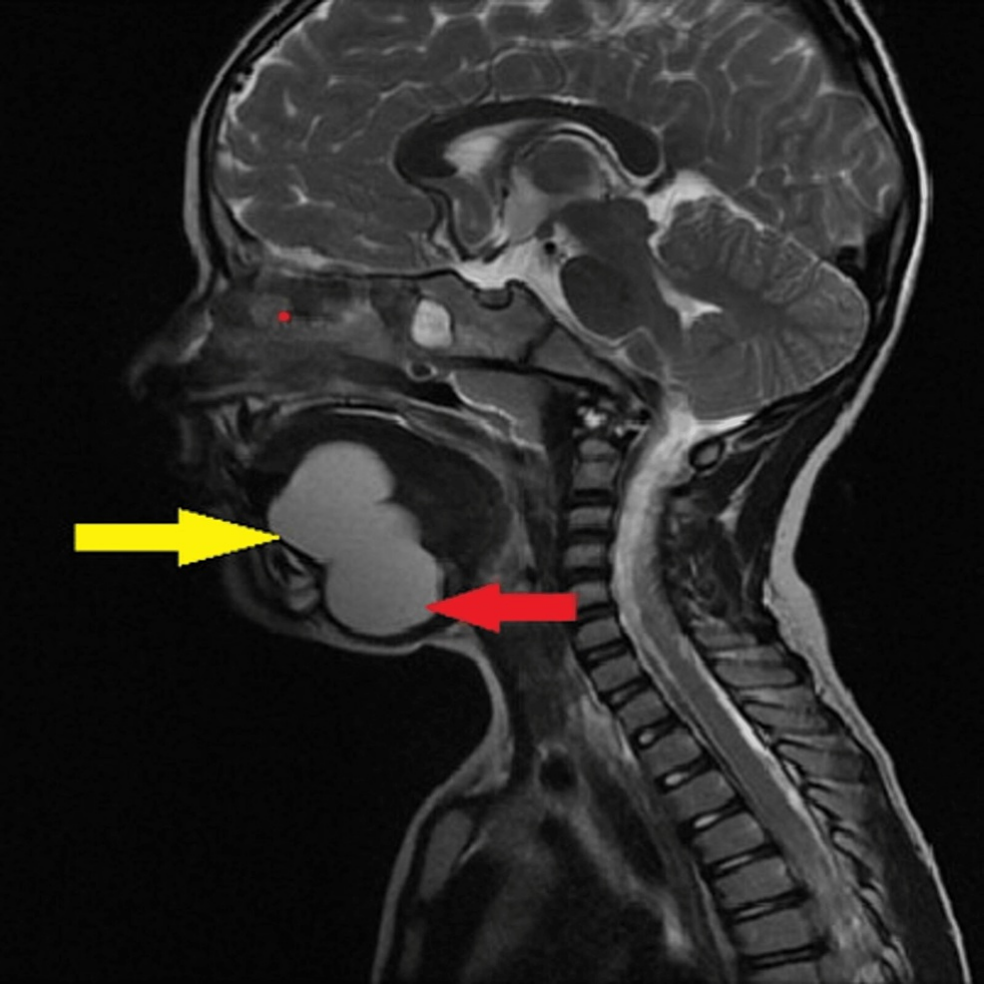
Sagittal T2 weighted image showing a well-defined hyperintense lesion with a sublingual component (yellow arrow) and a submandibular component (red arrow).

**Figure 7:**
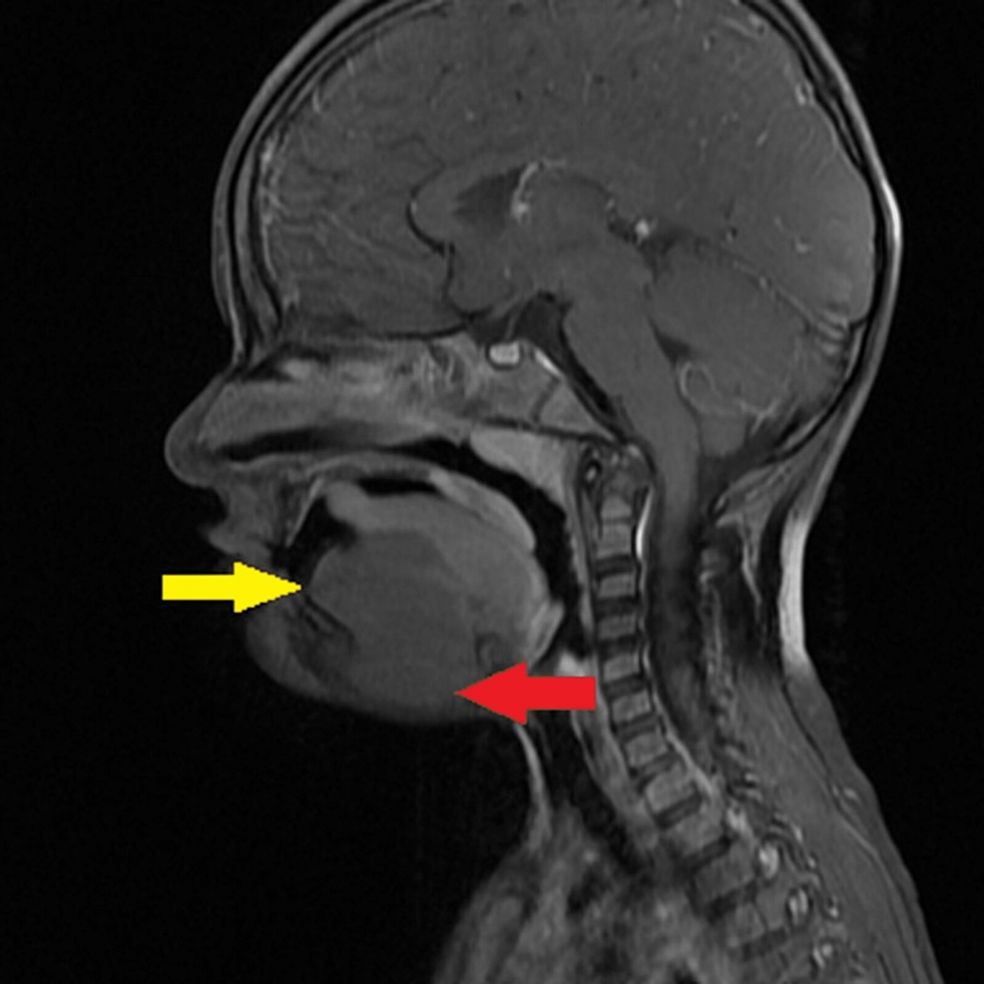
Sagittal postcontrast T1 weighted image showing no postcontrast enhancement in the lesion.

A well-defined lesion measured 2.4 x 2.8 x 4.7 cm (volume 16cc) in sublingual and submandibular regions. The lesion is iso-hypointense on T1WI and hyperintense on T2Wl and STIR sequences. No postcontrast enhancement is present (Figures 5-7).

The patient was subsequently operated, and the diagnosis of a plunging ranula was confirmed.

## Discussion

Plunging ranulas usually present as a painless cystic swelling located in the sublingual and submandibular regions. They are caused due to an obstruction in the duct of the sublingual gland. They extend or plunge beyond the mylohyoid muscle to enter the submandibular space. They are rare oral cystic lesions with a prevalence of 0.2 cases per 1000 individuals [2].

On ultrasound, plunging ranulas are well-defined cystic lesions with thin walls and lack vascularity on color Doppler. Few thin septae and internal echoes may be present. A cystic lesion in relation to the sublingual gland and mylohyoid muscle is a classical ultrasound feature of plunging ranulas [5]. This case report emphasizes the importance of an intraoral ultrasound approach for better visualization of the sublingual component of the ranula. On MRI, plunging ranulas are shown as iso-hypointense in the T1 weighted Image and hyperintense in the T2 weighted image and STIR sequences [6]. Usually, there is no postcontrast enhancement; however, enhancement of the capsule may be seen, if there is a secondary infection. On CT, plunging ranulas are seen as well-defined lesions with a smooth capsule and a fluid density component within them without septations. Cross-sectional imaging shows the extension and also shows the presence of ectopic sublingual glands [7].

A common complication of a large ranula is difficulty in speaking and eating. The patient in this case complained of difficulty in speech due to elevation of the tongue by the sublingual component of the ranula. A large ranula can cause tracheal compression and undergo rupture [8]. Common differential diagnosis of plunging ranulas is cystic hygroma, second branchial cleft cysts, dermoid cysts, epidermoid cysts, and thyroglossal cysts [1]. Ranulas are ideally managed by excision of the sublingual gland intraorally. If managed by only drainage of the cyst, recurrence is common. Postoperative complications include a tingling sensation in the tongue, hematoma, and local infection [9]. 

## Conclusions

Ranulas are cystic intraoral swelling located in the floor of the mouth. Plunging ranulas extend or plunge beyond the mylohyoid muscle and enter the adjacent submandibular or parapharyngeal space. Imaging of a plunging ranula is a little complex due to its location and components. Ultrasound and magnetic resonance imaging play a crucial role in the early diagnosis as well as planning an appropriate surgical approach and avoiding complications.
